# Low test–retest reliability of a protocol for assessing somatosensory cortex excitability generated from sensory nerves of the lower back

**DOI:** 10.3389/fnhum.2022.898759

**Published:** 2022-08-23

**Authors:** Katja Ehrenbrusthoff, Cormac G. Ryan, Denis J. Martin, Volker Milnik, Hubert R. Dinse, Christian Grüneberg

**Affiliations:** ^1^Department of Applied Health Sciences, Hochschule für Gesundheit, Bochum, Germany; ^2^School of Health & Life Sciences, Teesside University, Middlesbrough, United Kingdom; ^3^Neurological Clinic, Düren, Germany; ^4^Department of Neurology, Berufsgenossenschaftliches Universitätsklinikum Bergmannsheil GmbH, Ruhr-University Bochum, Bochum, Germany; ^5^Institute for Neuroinformatics, Neural Plasticity Lab, Ruhr-University of Bochum, Bochum, Germany

**Keywords:** cortical excitability, cortical disinhibition, test–retest reliability, somatosensory evoked potentials, paired-pulse behavior

## Abstract

In people with chronic low back pain (CLBP), maladaptive structural and functional changes on a cortical level have been identified. On a functional level, somatosensory cortical excitability has been shown to be reduced in chronic pain conditions, resulting in cortical disinhibition. The occurrence of structural and/or functional maladaptive cortical changes in people with CLBP could play a role in maintaining the pain. There is currently no measurement protocol for cortical excitability that employs stimulation directly to the lower back. We developed a protocol for the measurement of single pulse somatosensory evoked potential (SEP) waveforms and paired-pulse behavior (PPB) generated from sensory nerves of the lower back and quantified its test–retest reliability in a sample of 30 healthy individuals to gain insights into the normal variability of cortical responses, which could then be compared to results from people with CLBP. We investigated cortical excitability by measuring SEPs and PPB. PPB was defined as the ratio of the amplitude of the second cortical response (A2s) divided by the first cortical response (A1). A2s was determined by subtracting the response to single-pulse stimuli from the paired pulse stimuli response to account for linear superposition effects. The test–retest reliability of the protocol was very poor with no evidence of systematic bias but a high amount of random variability between sessions. There was no significant difference in the right side PPB for session 1 (Mean ratio A2s/A1 = 0.66, SD = 0.54) and session 2 (Mean ratio A2s/A1 = 0.94, SD = 1.56); mean session difference [(95% CI) = −0.44 (−1.23 to 0.34); *t* (22) = −1.17, *p* = 0.26]. The ICC_3_._1_ (absolute agreement) for the outlier-removed right side PPB were 0.19 (95% CI: −0.84 to 0.66) and 0.43 for left side PPB (95% CI: −0.37 to 0.76). This finding potentially has wider implications for PPB protocols. If these findings were replicated in other groups and other nerves, it would question the validity of this measure more generally. However, these findings are restricted to healthy people and sensory nerves of the lower back and may not be generalizable.

## Introduction

Low back pain (LBP) is a major global health issue (Buchbinder et al., 2018). Rarely, a specific cause or nociceptive origin of LBP can be identified. Hence, the majority of LBP is defined as “non-specific” (Balagué et al., 2012). 10–15% of patients with an acute episode of LBP develop symptoms lasting longer than 3 months, defined as “chronic low back pain (CLBP)” (Balagué et al., 2012).

A mounting body of neuroscientific evidence has shown that individuals with chronic pain have alterations in their nervous system encoding and/or processing of sensory information (Apkarian et al., 2013).

Against this background, chronic pain can be understood as a result of maladaptive central-nervous system activity on a spinal, sub-cortical and cortical level, leading to neurochemical, structural and functional changes (Flor and Stolle, 2018).

In patients with CLBP, such maladaptive cortical level changes have been identified (Flor et al., 1997; Apkarian et al., 2004; Moseley and Flor, 2012). On a functional level, the cortical representation of the lower back in the primary somatosensory cortex (S1) has been shown to be different in people with CLBP compared to healthy individuals (Kregel et al., 2015). A further feature of functional adaptation is cortical excitability (David-Jürgens and Dinse, 2010). The cortex ability to suppress afferent information that follows in close succession to an earlier incoming signal is one critical feature for precise neural activity encoding. In addition, it was shown that receptive fields are kept small by means of active intracortical inhibition (Hicks and Dykes, 1983). Both features represent aspects of the construct of cortical inhibition (Kalisch et al., 2009). Cortical inhibition properties have been shown to be reduced in older people (Dinse et al., 2006) and people with chronic pain conditions, resulting in the maladaptive equivalent, termed “cortical disinhibition” (Lim et al., 2015).

Cortical excitability can be investigated *via* the measurement of somatosensory evoked potentials (SEPs) and paired-pulse behavior (PPB). This is a technique to investigate changes in, and the balance between, cortical excitation and inhibition. Here, the term “paired-pulse suppression (PPS)” describes the phenomenon that at short interstimulus intervals (ISIs) neuronal responses (derived from S1) to the second stimulus are significantly reduced. PPS is quantified in terms of the ratio of the amplitude of the second response divided by the first. That means that a large ratio (≥1) is associated with cortical excitation or facilitation, and a small amplitude ratio (≤1) is associated with PPS or cortical inhibition (David-Jürgens and Dinse, 2010).

The co-occurrence of structural and/or functional maladaptive cortical changes in people with CLBP, termed as cortical reorganization, could constitute an obstacle to successful recovery (Moseley and Flor, 2012).

Clinically, cortical reorganization in patients with CLBP entails a disrupted body perception (Wand et al., 2011), which presents in various different ways and is frequently termed as “sensorimotor dysfunction,” mirroring the interweaving of altered motor behavior, and/or the distorted interpretation or inaccurate input of afferent sensory information (Hodges and Falla, 2015; Pelletier et al., 2015). Goossens et al. (2018) proposed that, as adequate sensory processing and integration are indispensable for optimal motor control, a maladaptive encoding and/or processing of sensory information may disturb spinal and postural control in patients with CLBP. Hence, the processing and interpretation of sensory input derived from the area concerned needs to be investigated in people with CLBP (Brumagne et al., 2019).

The PPB of peripheral nerves, such as the median nerve, have previously been investigated in a number of different pain populations, e.g., in patients with complex regional pain syndrome (CRPS) (Lenz et al., 2011, 2013) and fibromyalgia (FM) (Lim et al., 2015). Lenz et al. (2011) found bilaterally disturbed cortical inhibition properties in people with CRPS I and further assumed that these changes in cortical excitability could constitute a predispositional factor for developing CRPS. In people with FM, Lim et al. (2015) similarly found a bilaterally compromised intracortical inhibition in S1 compared to healthy controls and interpreted these differences as a potential contributing factor to the pathophysiology of FM pain.

As yet, no study has investigated PPB in people with CLPB, although similar structural and functional cortical changes may occur in this pain population.

Against the above-mentioned considerations, several methodological requirements needed to be investigated to explore the usability of the PPB measurement approach in people with CLBP. Firstly, the sensory information should ideally be derived from nerves of the area concerned, i.e., the lower back to gain insights into potentially maladaptive processing and interpretation of sensory information stemming from that region. This consideration ruled out the use of already established measurement protocols, such as for the median or tibial nerve (Onishi et al., 1991).

Secondly, previous research demonstrated that PPB, induced by somatosensory stimulation, varied considerably, even among healthy young individuals (Lenz et al., 2012; Onishi et al., 2016) although a clear reason for the high variability within and between subjects is hitherto not fully understood (Onishi et al., 2018). Therefore, the test–retest reliability of our PPB measurement protocol needed to be quantified in healthy individuals before using it in people with CLBP.

Against this background, the aim of this study was to develop a protocol for the measurement of single-pulse SEPs and PPB generated from sensory nerves of the lower back and to quantify its test–retest reliability in a sample of healthy individuals. If our measurement protocol revealed that PPB assessments varied between sessions even in healthy people, the statistical power of these measurements would be decreased. This again would subsequently limit the robustness of conclusions drawn regarding the differences between healthy controls and people with CLBP or the effects of treatments administered to this pain population. However, the test–retest results generated from the single-pulse and paired-pulse stimulation of the sensory nerves of the lower back needed to be backed up against potential confounding variables. To ensure this integrity of the complete somatosensory lemniscal conduction pathways, we decided to add the measurement of sural nerve SEPs to the overall measurement protocol. As normative values for this sensory nerve of the lower limb were already published by different laboratories (Tackmann, 1993; Maurer, 2005), the data from this study sample could be compared to them. With this approach, we aimed to detect potentially hidden abnormalities regarding the conduction velocity of the SEPs, which could potentially influence the results generated from the stimulation of the lower back cutaneous sensory nerves.

## Material and methods

In this study, 30 healthy individuals were assessed during two independent sessions by means of a standardized SEP measurement protocol. Session 2 took place between 1 and 7 days later with the same protocol repeated as for session 1.

The reporting of this study followed the Guidelines for Reporting Reliability and Agreement Studies (GRRAS) (Kottner et al., 2011) (see Supplementary material). The reporting of statistical results was further guided by the “Improved Statistical Analyses and Methods in the Published literature (SAMPL) guidelines” (Indrayan, 2020).

### Participants

A convenience sample of 30 healthy volunteers were recruited from staff, family/friends of staff, and students of the Hochschule für Gesundheit, Bochum (University of Applied Sciences). Participants had to be between 18 and 50 years old. Fifty years was considered to be sufficiently low not to demonstrate any variations in SEPs/PPB associated with aging (Lenz et al., 2012). Participants needed to have sufficient cognitive and German language ability to understand both oral and written instructions and provide informed consent. They needed intact skin on the lower back. In addition, they needed to have a successful thermal skin sensation test of the feet to allow for an assessment of the sural nerve.

Participants were excluded if they reported known or diagnosed neurological diseases or showed “red flags” indicating serious spinal or peripheral nerve root pathologies or signs of malignancy, fracture, infection, inflammatory joint or bone disease. Participants were excluded if they reported back or lower limb pain, currently or within the previous year. Previous surgery with metal implants in the trunk or pelvis, pregnancy and cardiac pacemaker were further exclusion criteria as they constituted contraindications for electrical stimulation of the trunk (Stöhr et al., 2013). In addition, participants were excluded when they reported a communicable foot condition or current (or history of) altered thermal sensation of either foot, as this was an essential prerequisite for the assessment of the sural nerve.

The following demographics were recorded; age, gender, height, weight, BMI, and hand dominance. This study aimed to recruit 30 participants. The sample size considerations for this study were based upon recommendations from the American Clinical Neurophysiology Society and current textbooks on electrophysiology diagnostics (American Clinical Neurophysiology Society, 2006b; Buchner, 2015).

The study was approved by Teesside University’s School of Health and Social Care Research Governance and Ethics Board (Study No. 015/16) and the Ethics committee of the German National Physiotherapists Society.

The study was registered in the German Clinical Trials Register (Registration number: DRKS00015109).

### Setting

Participants were investigated in a laboratory-based setting in the Hochschule für Gesundheit. The average room temperature was 24.5°C. A comfortable room temperature is important as a low limb temperature can prolong the absolute latency of cortical SEPs (Watson and Carter, 2016).

### Somatosensory evoked potential measurement protocol

To record the cortical SEPs generated from stimulation of the lower back cutaneous sensory nerves, the electrode placement was chosen according to the international ten-twenty electrode placement system for EEG recordings, with the recording electrode at CPZ and the reference electrode at FZ (Klem et al., 1999). This configuration was adopted from recommendations for SEP recordings following lower limb stimulations (Chiappa, 1997c), as the cortical topographic maps of the lower limb and back were shown to be indiscernible and located on the margo superior cerebri (Feldman and Brecht, 2005; Jurcak et al., 2007; Harding-Forrester and Feldman, 2018). A grounding electrode was placed at the wrist to be placed midway between stimulation and recording site (Daube and Rubin, 2014).

**FIGURE 1:**
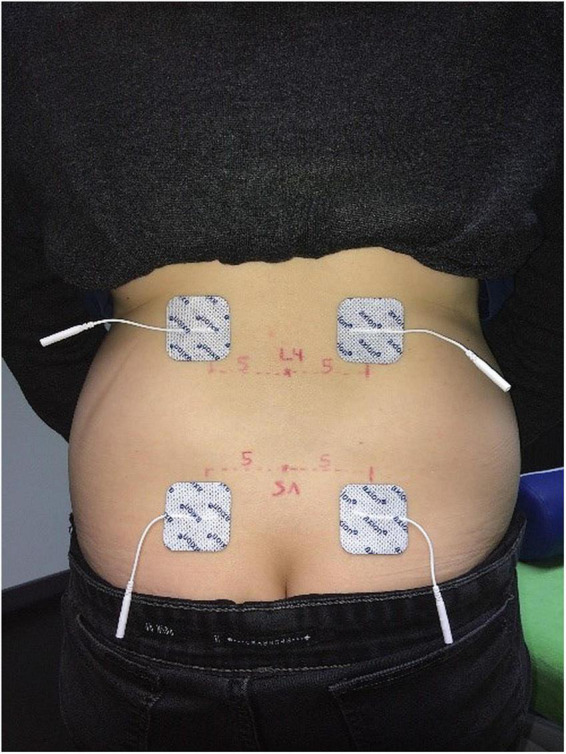
Stimulation electrode position.

In addition, two sets of disposable self-adhesive stimulation electrodes (40 × 40 mm, Axion, Leonberg, Germany) were placed on either side of the back to consecutively stimulate the left and right side of the lower back and to record cortical answers separately. So, measurements could be obtained from both sides of the back, but participants did not have to change their position during measurements. A separate measurement for left and right side, respectively was chosen to obtain insights into potential differences between cortical answers from different stimulation sides. This information could be used for the interpretation of results from patients with unilateral back pain, as to whether one side response exceeded the normal side to side differences measured in healthy individuals, In a standardized palpation procedure (Merz et al., 2013) the spinal process of L4 was located and marked by a washable pen. The upper stimulation electrodes were placed on the upper edge of the L4 level, five cm apart from the midline. The lower electrodes were placed 10 cm below. With this set-up a sufficiently broad area was covered to account for the lower cutaneous sensory fiber density at the lower back (Mauguière and Garcia-Larrea, 2017). See Figure 1 for illustration.

The measurement of the sural nerve was realized by using the following approach: prior to the actual measurement and to account for potential thermal skin sensation disturbances a thermal skin sensation test was conducted by means of a reliable and valid thermoception device (Windecker et al., 1997) (TIP THERM^®^, tip therm GmbH, Dorsten, Germany). The skin temperature of both lower limbs needed to be above 34°C (Joerg, 1983). A skin temperature below 30°C would artificially prolong the absolute latency, as both features are linearly correlated (Markand et al., 1990). To ensure a sufficiently high skin temperature, participants were asked to bath their feet for 5 min in a tub with warm water (35°C) reaching up over the inner and outer malleolus before the measurement of the sural nerve. The recording block electrode was positioned successively at both lateral malleoli with the cathode placed proximal in an orthodromic positioning.

### Somatosensory evoked potential recording

All recordings were undertaken with a Neuropack MEB-2300 EMG/EP Measuring System (Version 04-03, Nihon Kohden, Tokyo, Japan). Participants lay on a bench in a quiet, darkened and electrically shielded room. They were instructed to keep their eyes closed and to relax during all recordings. They were asked to count from 0 to 100 during the recordings to facilitate a constant vigilance.

### Order of stimulation conditions

Firstly, the single-pulse SEP stimulation protocol was performed with successive stimulation of the right and left side of the lower back. After this, the measurement of the sural nerve was undertaken to allow the lower back a recovery phase of approximately 10 min from electrical stimulation. Thereafter, the paired-pulse stimulation protocol was applied with successive stimulation of the right and left lower back.

Firstly, single-pulse SEPs were recorded successively in response to stimulation of the lower back with the cathode placed proximally. Electrode impedances were kept below 5 kiloohm (kΩ) throughout the session. Electrical stimuli consisted of continuous single square wave pulses [0.1 ms duration per pulse delivered at a constant frequency of 2.9 Hertz (Hz)]. The SEPs were recorded in epochs from 0 to 100 ms after stimulus onset. Five hundred stimulus-related SEP signals were recorded at a time and averaged to one resulting trace. The upper frequency limit was set at 1 kilohertz (kHz), the lower frequency limit at 1 Hz. The cortical waveforms of the single-pulse SEPs were analyzed regarding latencies of responses from stimulus to initial positive peak (P1) and amplitude of waveforms from initial peak of one polarity [initial negative (N1)] to the immediately following peak of opposite polarity [initial positive peak (P1)] (Chiappa, 1997b).

Prior to the recordings, the sensory threshold was determined individually by manually increasing the stimulator current output in increments of 0.2 milliampere (mA). The sensory threshold was defined as the lowest level of electrical stimulus intensity that produces the subtle tactile sensation on the skin of the lower back. This electrical stimulus intensity was multiplied by three, as this has previously been defined to be an adequate stimulation intensity for pure sensory nerves (Hoeffken et al., 2013). The sensory threshold was determined anew for the second session and the stimulation intensity was set accordingly.

### Paired-pulse stimulation

To assess PPB, a paired-pulse protocol consisting of innocuous paired electrical stimulation of the lower back cutaneous sensory nerves with a stimulus onset asynchrony (SOA) of 50 ms was applied with 0.2 ms duration per pulse delivered at a constant frequency of 2.9 Hz. The ISI of 50 ms was chosen, as provisional data from rehearsals of process showed, that the commonly applied ISI of 30 ms, used in paired-pulse stimulation protocols for the median nerve (Dinse et al., 2006; Puta et al., 2016; Enax-Krumova et al., 2017) interfered with the first cortical response. In addition, normal values for latencies from SEPs generated from the lower back did not exist. Hence, the ISI of 50 ms appeared to be sufficiently wide to ensure an undisturbed first cortical response.

Peak-to-peak amplitudes of the cortical N1–P1 response component for the first and second paired-pulse stimulus (with the peak-to-peak amplitude of N2-P2) were analyzed. After nine participants were assessed following this approach, it became apparent that the first cortical signal could be expected at approximately 30 ms after stimulation. Hence, the ISI of 50 ms was considered to be potentially unnecessarily wide to measure PPB. Hence, the reduction of the ISI to 40 ms was introduced as an additional measurement step to independently quantify the PPB at both 50 ms and 40 ms. Between both stimulation protocols, the participant had a resting phase of approximately 5 min. Hoeffken et al. (2013) showed that in studies of PPB after stimulating the median nerve in healthy people, a significant suppression of the second cortical response was shown for short ISIs of 20 to 40 ms. For ISIs exceeding 100 ms, no significant suppression could be observed. Hence, by comparing an ISI of 40 and 50 ms it could be explored which ISI would best be fitting for assessing PPB generated from the lower back, with both ISIs well below the threshold of 100 ms, where no cortical suppression could be expected. This approach was explored in 20 out of the 30 participants.

**FIGURE 2:**
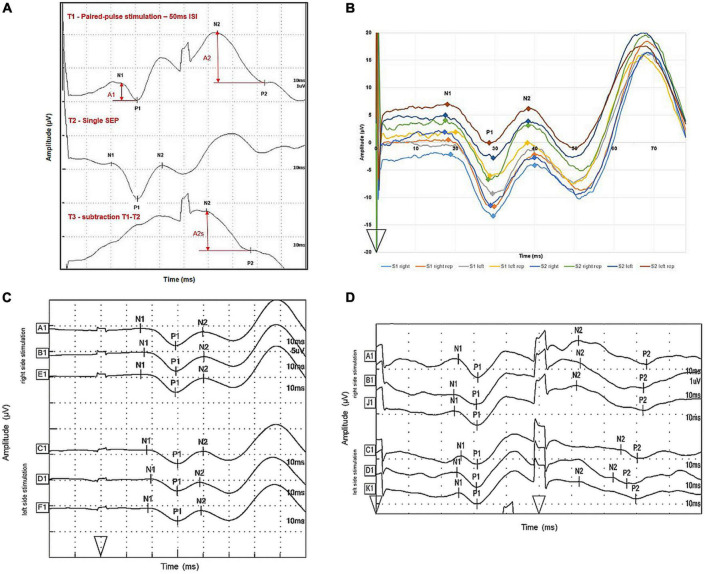
Illustration of SEP trace subtraction procedures and intra-individual variability of one subject. **(A)** Evoked potentials were measured over cortical CPZ during single-pulse (middle trace) and paired-pulse (upper trace) stimulation of right lower back cutaneous sensory nerves. The bottom trace shows the results by subtracting the single-pulse trace from the paired-pulse trace. The analyzed amplitudes of the first response (A1) and second response (A2) after paired-pulse stimulation are marked by vertical arrows; amplitudes of the second response after subtracting the response to a single pulse are denoted as A2s. Onset of stimulation is marked by arrowheads. One unit on the *x*-axis represents 10 ms, one unit on the *y*-axis represents 1 μV. **(B)** Evoked potentials were measured over cortical CPZ after successive single-pulse stimulation of right and left lower back cutaneous sensory nerves. S1 right is the trace for right side stimulation of the lower back in session 1, S1 right rep stands for waveform, meeting the criteria for sufficient replication. S1 left and S1 left rep were originally recorded after left side stimulation in session 1. S2 right and S2 right rep stand for the same procedure in session 2 and S2 left and S2 left rep for left side stimulation in session 2, respectively. Onset of stimulation is marked by an arrowhead. One unit on the *x*-axis represents 10 ms, one unit on the *y*-axis 10 μV. **(C)** Evoked potentials were measured over cortical CPZ after successive single-pulse stimulation of right and left lower back cutaneous sensory nerves. A1 and B1 were traces meeting the criteria of sufficient replication. E1 is the average trace out of A1 and B1. C1 and D1 were originally recorded after left side stimulation; F1 is the average trace out of C1 and D1. The markers N1, P1, and N2 from trace E1 and F1 were used for calculating descriptive data. Onset of stimulation is marked by an arrowhead. One unit on the *x*-axis represents 10 ms, one unit on the *y*-axis represents 5 μV. **(D)** Evoked potentials were measured over cortical CPZ after successive paired-pulse stimulation of right and left lower back cutaneous sensory nerves. A1 and B1 were traces meeting the criteria of sufficient replication. J1 is the average trace out of A1 and B1. C1 and D1 were originally recorded after left side stimulation; K1 is the average trace out of C1 and D1. The traces J1 and K1 were used for subtraction. Onset of stimulations are marked by arrowheads. One unit on the *x*-axis represents 10 ms, one unit on the *y*-axis represents 1 μV.

As illustrated for one participant in Figure 2A, after paired-pulse stimulation the response to the second pulse rides on the response to the first pulse, leading to a superposition of both evoked potentials. Following the recommendations from Mauguière and Garcia-Larrea (2017), linear superposition effects were factored out by subtracting the response to the single-pulse stimulation from the paired-pulse stimulation trace. The second paired-pulse SEP amplitude was defined after linear subtraction of the single-pulse trace SEP (A2s) and was referenced to as the first paired-pulse SEP amplitude before linear subtraction (A1). PPS was expressed as a ratio (A2s/A1) of the amplitudes of the second (A2s) and the first (A1) peak. See Figure 2A for details.

### Data processing

All recordings were replicated at least once to demonstrate that the waveforms were consistently repeatable and therefore of neural and not artifactual origin. Replication was defined to be demonstrated by the consistency of the first positive peak (P1) within 0.5 ms in successive averages (American Clinical Neurophysiology Society, 2006b). Two separate traces, which met the replication criteria, were then averaged. Within this averaged trace, markers were set manually to determine the cortical peaks, in keeping with the recommendations of international SEP guidelines (American Clinical Neurophysiology Society, 2006a). Amplitudes were automatically calculated as potential difference between cortical peaks of opposite polarity by the Neuropack software. Waveforms, latencies and amplitudes were stored on the Neuropack device for offline analysis and transferred to Microsoft Excel 2016 (Microsoft, Redmond, WA, United States) and SPSS, version 24 (IBM, Armonk, NY, United States) for further analysis.

### Statistics

In order to comprehensively describe values for single SEPs and PPB, a variety of different measures of central tendency and spread were calculated. The mean, standard deviation (SD), median, and interquartile ranges (IQR) for N1, P1, N2, and P2 latencies as well as for N1/P1 and N2/P2 amplitudes were reported. In addition, side-to-side differences (STSD) for these latencies were reported by subtracting right side from left side values. To calculate the side-to-side asymmetry ratios (STSAR) of N1/P1 and N2/P2 amplitude and for the PPB, expressed as the ratio (A2s/A1), the following equation was used (Moncho et al., 2015):


S⁢T⁢S⁢A⁢R=|(L⁢e⁢f⁢t-R⁢i⁢g⁢h⁢t)|⁢/⁢[(L⁢e⁢f⁢t+R⁢i⁢g⁢h⁢t)⁢/⁢2]

All ratios were expressed as arbitrary units.

To explore the difference between PPB of 40 and 50 ms ISIs, amplitude ratios (A2s/A1) from the first session were compared using a paired *t*-test for the 20 participants who underwent the 50 and 40 ms ISI measurement protocol.

Test–retest reliability was assessed using a battery of measures to quantify both random error and systematic error, concentrating on the values of PPB (A2s/A1 amplitude ratios) as this parameter is indicative of the cortical inhibition properties under investigation. Here, change scores between session 1 and 2 regarding A2s/A1 ratios were used for assessing the assumption of normal distribution. For this purpose, histograms, Q–Q and box plots were assessed for normal distribution by visual inspection rather than discrete statistical testing (Field, 2013; Rani Das and Imon, 2016). Outliers were identified according to the “inter-quartile range rule” by visual inspection of the box plots where values of more than three IQRs from the end of the box were labeled by an asterisk and values more than 1.5 IQRs but less than 3 IQRs with a dot (Hoaglin and Iglewicz, 1987).

Random error between sessions was quantified with the within-subjects SD [standard error of measurement (SEM)], coefficient of variation (CV), limits of agreement (LOA), and a random-error only (model 3.1) intraclass correlation coefficient (ICC). ICC_3_._1_ scores of <0.75 were considered to demonstrate poor reliability, 0.75–0.89 moderate and ≥0.90 excellent reliability (Portney and Watkins, 1993). Reliability results were visualized by means of Bland-Altman Plots. The mean, SD systematic bias (and associated 95% confidence interval) between data collected at session 1 and 2 was quantified using a paired *t*-test.

## Results

### Participant characteristics

Thirty people enquired about the study of which all 30 met the inclusion criteria and consented to participate. All participants completed the study. The participant characteristics for the 30 healthy people are detailed in Table 1.

**TABLE 1:** Participant characteristics for *n* = 30 healthy people.

	*N*	Mean	SD	Minimum	Maximum
**Demographic data**					
Age (years)	35	30.7	7.1	21	47
Gender	7 (♂)23 (♀)	n.a.	n.a.	n.a.	n.a.
Height (m)	30	1.7	0.1	1.6	1.9
Weight (kg)	30	68.8	14.0	51.0	105.0
Body Mass Index (kg/m^2^)	30	23.2	4.0	17.2	36.3
Handedness	28 right1 left1 amb.	n.a.	n.a.	n.a.	n.a.

SD, standard deviation; amb, ambidextrous; n. a., not applicable.

In this study, it was decided that Handedness was established by enquiring about preferred hand while performing everyday tasks.

### Single-pulse somatosensory evoked potential waveforms

Figure 2B illustrates a typical single-pulse SEP waveform generated within one subject after right and left stimulation of the lower back for session 1 and 2, respectively to illustrate the amount of intra-individual variability of the traces. Figure 2C then illustrates the further processing of the average traces for right and left side stimulation. Table 2 summarizes the descriptive statistics of values separated for right and left side stimulation of lower back cutaneous sensory nerves.

**TABLE 2:** Descriptive statistics for latencies, amplitudes and side-to-side differences for single-pulse SEPs after cutaneous sensory nerve stimulation of the lower back.

			Mean	SD	Median	IQR	Range
Latencies (ms)	Right side stimulation	N1	19.56	3.05	18.55	17.83 to 20.4	14.80 to 27.20
		P1	29.92	2.61	30.40	29.23 to 31.30	22.00 to 34.00
		N2	39.15	3.19	39.20	38.4 to 40.60	25.10 to 44.70
	Left side stimulation	N1	19.68	2.70	19.35	18.18 to 20.50	15.90 to 27.70
		P1	30.54	1.78	30.65	29.93 to 31.70	26.10 to 33.40
		N2	39.53	3.32	40.30	37.68 to 41.10	28.90 to 44.20
Amplitudes (μV)	Right side stimulation	Pair 1 (N1/P1)	0.75	0.65	0.61	0.34 to 0.96	0.07 to 3.48
		Pair 2 (P1/N2)	0.75	0.59	0.59	0.40 to 0.94	0.05 to 2.73
	Left side stimulation	Pair 1 (N1/P1)	0.76	0.57	0.59	0.36 to 1.09	0.10 to 2.46
		Pair 2 (P1/N2)	0.80	0.51	0.71	0.42 to 1.07	0.12 to 1.99
Latencies (ms)	Side-to-side differences (STSD)	N1	0.12	3.03	0.25	−1.75 to 1.80	−5.50 to 7.30
		P1	0.62	2.01	0.35	−0.30 to 1.10	−3.30 to 6.40
		N2	0.37	4.19	0.50	−1.5 to 2.10	−10.00 to 15.80
Amplitudes	Side-to-side asymmetry ratios (STSAR) of amplitudes	Pair 1 (N1/P1)	0.42	0.38	0.38	0.13 to 0.52	0 to 1.60
		Pair 2 (P1/N2)	0.39	0.39	0.29	0.08 to 0.24	0.15 to 0.49

IQR, interquartile range; ms, milliseconds; μV, microvolt.

### Sural nerve data

The data from session 1 were used to determine the sural nerve SEP descriptive values. The data provided evidence of the integrity of the complete somatosensory lemniscal conduction pathways and the values obtained were in keeping with previous normative data (Eisen and Elleker, 1980; Tackmann, 1993), which adds confidence to the testing procedure used in this study. Descriptive data of the sural nerve are presented as Supplementary material.

### Missing data

Missing data occurred in the first and second measurement session dues to several reasons. In session 1, no missing data were recorded for the single-pulse SEP and the sural nerve measurement. For the 50 ms ISI protocol in session 1, no second amplitude was determinable in three participants for left and two participants for right side stimulation. For the 40 ms ISI protocol in session 1, there were four participants for whom the second amplitude could not be determined for left and right side stimulation. In the further data processing, it occurred that second amplitudes (A2s) were no longer determinable after the subtraction procedure, because positive or negative peaks (N2 or P2) were no longer definable from the subtracted trace. In session 1, this applied for five participants in the 50 ms ISI protocol. As only complete data sets were included in the final analysis, 14 data sets could be used for the analysis of right side differences between the 40 and 50 ms ISI protocols and 13 for left side differences.

### Exploration of differences between 50 and 40 ms interstimulus interval paired-pulse behavior

A paired-samples *t*-test was conducted to compare the PPB using an ISI of 50 ms and an ISI of 40 ms for right and left side stimulation separately. There was no significant difference in the right side scores for 50 ms PPB, [expressed by amplitude ratios (A2s/A1)] (Mean = 0.71, SD = 0. 67) and right side 40 ms PPB (Mean = 1.89, SD = 3.11); mean session difference [(95% CI) = −1.18 (−2.99 to 0.63); *t* (13) = −1.41, *p* = 0.18]. There was no significant difference in the left side scores for 50 ms PPB, [expressed by amplitude ratios (A2s/A1)] (Mean = 0.76, SD = 0.72) and left side 40 ms PPB (Mean = 1.30, SD = 1.01); mean session difference [(95% CI) = −0.55 (−1.26 to 0.17); *t* (12) = −1.67, *p* = 0.12]. Thus, as there was little difference between both ISIs and as there were more data sets available from the 50 ms ISI protocol, it was decided to report the test–retest reliability data from the 50 ms ISI protocol.

### Paired-pulse behavior somatosensory evoked potential descriptive data

**FIGURE 3:**
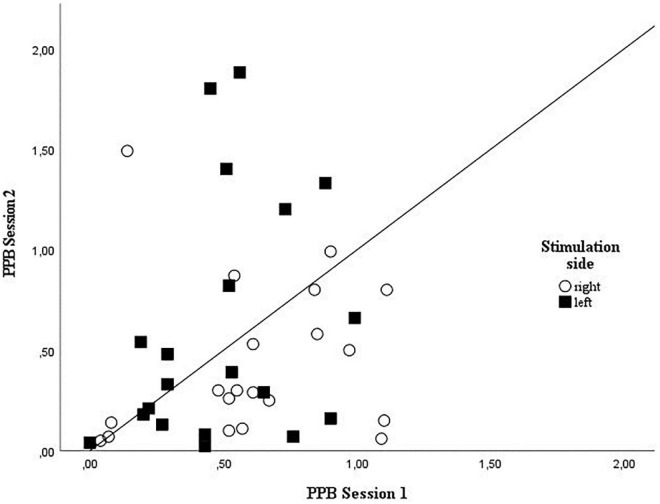
Grouped scatterplot showing the individual amplitude ratios for session 1 and 2 for right and left side stimulation. Right side stimulation amplitude ratios are plotted as circles, left side stimulation amplitude ratios squares values identified as outliers were excluded from plotting; PPB, paired-pulse behaviors.

The data from session 1 from the 50 ms ISI protocol were used to determine the PPB SEP descriptive values. After exclusion of incomplete data sets due to missing data, data sets from 23 participants could be used for further analysis. Figure 2D illustrates a typical SEP waveform generated after paired-pulse stimulation of the lower back with an ISI of 50 ms along with the processing of the average traces for right and left side stimulation. The average traces were then used for subtraction. Table 3 summarizes the descriptive statistics of SEP values separated for right and left side paired-pulse stimulation with an ISI of 50 ms. Figure 3 illustrates the distribution of amplitude ratios for session 1 and 2 separated for right and left side stimulation.

**TABLE 3:** Descriptive statistics for latencies, amplitudes and side-to-side differences for SEPS after paired-pulse cutaneous sensory nerve stimulation of the lower back with an ISI of 50 ms (session 1 data).

			Mean	SD	Median	IQR	Range
Latencies (ms)	Right side stimulation	N1	18.72	3.31	18.90	16.65 to 20.80	13.10 to 25.60
		P1	29.33	3.54	30.50	27.9 to 31.15	18.50 to 34.60
		N2	67.91	3.63	67.70	66.00 to 70.10	60.10 to 75.10
		P2	79.77	3.86	79.20	77.5 to 81.88	74.90 to 94.70
		N2s	71.12	5.20	70.20	68.55 to 74.50	60.80 to 81.50
		P2s	77.16	5.67	76.85	73.23 to 80.00	67.20 to 94.70
	Left side stimulation	N1	19.38	3.78	20.20	18.6 to 21.05	5.70 to 25.40
		P1	29.12	2.86	29.95	27.65 to 31.00	21.80 to 33.00
		N2	68.40	2.60	68.95	66.78 to 69.75	62.40 to 73.10
		P2	79.51	3.49	79.75	77.45 to 81.50	71.30 to 88.80
		N2s	70.68	5.01	70.30	68.1 to 73.83	60.50 to 80.50
		P2s	77.03	4.47	77.00	74.9 to 79.65	68.90 to 88.80
Amplitudes (μV)	Right side stimulation	Pair 1 (N1/P1)	0.61	0.56	0.49	0.31 to 0.65	0.11 to 3.14
		Pair 2s (N2s/P2s)	0.31	0.29	0.23	0.13 to 0.42	0.01 to 1.30
		PPB Ratio (pair 2s/pair 1)	0.66	0.54	0.56	0.43 to 0.88	0.02 to 2.65
	Left side stimulation	Pair 1 (N1-P1)	0.61	0.46	0.51	0.25 to 0.84	0.08 to 1.97
		Pair 2s (N2s-P2s)	0.32	0.28	0.26	0.14 to 0.42	0.00 to 1.36
		PPB (Ratio pair 2s/pair1)	0.67	0.59	0.52	0.29 to 0.77	0.00 to 2.88
Latencies (ms)	Side-to-side differences	N1	1.29	5.22	1.50	−1.1 to 3.38	−12.20 to 20.20
		P1	0.81	5.30	0.00	−1.9 to 1.93	−4.20 to 24.20
		N2s	−0.44	5.54	−1.80	−3.3 to 0.97	−7.10 to 13.60
		P2s	5.20	20.90	0.30	−1.60 to 3.28	−13.20 to 81.20
Amplitudes	Side-to-side asymmetry Ratios (STSAR)	Pair 1 (N1-P1)	0.57	0.47	0.51	0.22 to 0.82	0.00 to 2.00
		Pair 2s (N2s-P2s)	0.69	0.64	0.55	0.12 to 1.12	0.00 to 2.00

IQR, interquartile range; ms, milliseconds; μV, microvolt; N2s, N2 after subtraction; P2s, P2 after subtraction.

PPB (Ratio = A2s/A1) (expressed in arbitrary units).

### Reliability analysis

Upon visual inspection, the data for the PPB change scores (A2s/A1 amplitude ratios) between session 1 and 2 were not normally distributed for the right side values. The change data were markedly skewed to the right side, indicating a build-up of high change values. Left side values demonstrated sufficient normal distribution to proceed with the parametric analysis. For details of normality testing please see Supplementary material. To explore the effect of the non-normal distribution on the reliability analysis of the right sided PPB, sensitivity analyses were conducted to assess the robustness of the test–retest reliability findings according to the methodological approaches recommended by Thabane et al. (2013). Regarding the robustness of *t*-test results, the non-parametric equivalent, the Wilcoxon signed ranked test, was conducted. As to the potential influence of outliers on the random error component of the results, they were identified by the “IQR-rule,” defined by Field (2013) (any value greater than 3.0 × IQR + third quartile or any value below 3.0 × IQR − first quartile is defined as an outlier), and subsequently excluded from the analysis (Cousineau and Chartier, 2010).

### Test–retest reliability

A paired-samples *t*-test was conducted to compare the change scores PPB from session 1 to session 2 for right and left side stimulation separately. There was no significant difference in the right side PPB scores for session 1 (Mean = 0.66, SD = 0.54) and session 2 (Mean = 0.94, SD = 1.56); mean session difference [(95% CI) = −0.44 (−1.23 to 0.34); *t* (22) = −1.17, *p* = 0.26]. As a sensitivity analysis, the non-parametric equivalent, the Wilcoxon signed rank test was undertaken. This also indicated no difference between session 1 (Median = 0.56) and 2 (Median = 0.30) for right side PPB (*Z* = −0.76, *p* = 0.45). Hence, the findings of this sensitivity analysis support the findings of the primary analysis showing little evidence of systematic error between time 1 and 2 for PPB on the right side.

**FIGURE 4:**
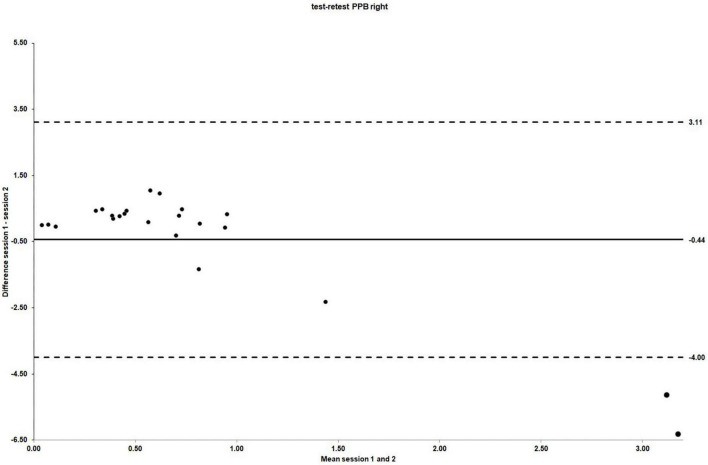
The LOA for PPB right side test–retest reliability. For test–retest reliability, test–retest differences are plotted against the pooled means from session 1 and 2. Mean session differences (systematic bias) are displayed by solid lines and LOA by dashed lines.

The ICC_3_._1_ values for absolute agreement were −0.25 for right side PPB (95% CI: −1.91 to 0.47), indicating no agreement between sessions (Giraudeau, 1996; Wirtz and Caspar, 2002). The Bland and Altman plot for the individual differences for right side PPB between session 1 and 2 is shown in Figure 4. Table 4 summarizes the test–retest reliability data for the right side PPB along with the associated 95% CIs.

**TABLE 4:** Comparison of PPB test–retest reliability results between sides.

	Test–retest reliability right side PPB	Test–retest reliability right side PPB–outliers removed	Test–retest reliability left side PPB
Mean session difference (95% CI)	−0.44 (−1.23 to 0.34)	0.18 (−0.05 to 0.41)	−0.07 (−0.46 to 0.31)
SD of session differences (95% CI)	1.81 (1.39 to 2.62)	0.48 (0.37 to 0.71)	0.89 (0.68 to 1.28)
Within-subjects SD (SEM) (95% CI)	1.28 (0.98 to 1.85)	0.34 (0.26 to 0.50)	0.63 (0.48 to 0.90)
Coefficient of variation (%) (95% CI)	161.04 (123.21 to 232.56)	63.65 (47.76 to 95.42)	88.51 (67.71 to 127.81)
Limits of agreement (LOA) (95% CI)	3.55 (2.72 to 5.13)	0.95 (0.71 to 1.42)	1.74 (1.33 to 2.51)
ICC_3_._1_ (95% CI)	−0.25 (−1.91 to 0.47)	−0.19 (−2.17 to 0.53)	0.43 (−0.37 to 0.76)

SD, standard deviation; SEM, standard error of measurement; CI, confidence interval, ICC_3_._1_, intraclass correlation coefficient (absolute agreement).

The values for the 95% Confidence Intervals were calculated according to the recommended method by Zar (1999).

**FIGURE 5:**
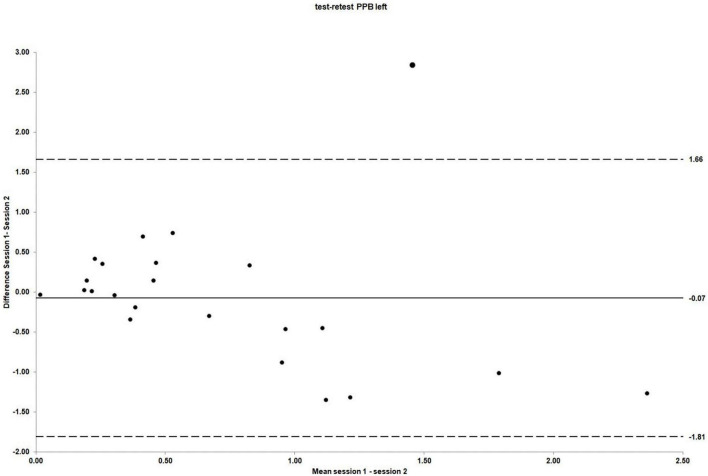
The LOA for PPB left side test–retest reliability. For test–retest reliability, test–retest differences are plotted against the pooled means from session 1 and 2. Mean session differences (systematic bias) are displayed by solid lines and LOA by dashed lines.

The mean scores for left side PPB obtained in session 1 and 2 were 0.67 (SD = 0.59) and 0.74 (SD = 0.80), respectively [mean difference −0.07 (95% CI: −0.46 to 0.31); *t* (22) = −0.39, *p* = 0.70]. As for the right side PPB, there was no evidence of systematic bias. The ICC_3_._1_ values for absolute agreement were 0.43 (95% CI: −0.37 to 0.76), indicating poor agreement between sessions (Portney and Watkins, 1993). The Bland and Altman plot for the individual differences for left side PPB between session 1 and 2 is shown in Figure 5.

As plotting the difference between session 1 and 2 revealed a significantly skewed distribution to the right for the right side PPB change scores, indicating a non-normal distribution of the data, a further sensitivity analysis for the random error components of the reliability analysis was conducted in that the outliers, identified by the “IQR-rule.” After removal of cases 1, 9, and 12, the distribution of the data shifted toward a more normal distribution. See Supplementary material for details. After the outliers were removed from the original reliability analysis, the results for the random error components improved as would be expected when removing those values with the greatest amount of variability; however, a considerable amount of random error persisted. The mean values for the outlier-removed right side PPB obtained in session 1 and 2 were 0.61 (SD = 0.34) and 0.46 (SD = 0.50), respectively [mean difference 0.18 (95% CI: −0.27 to 0.39, *p* = 0.70)]. The ICC_3_._1_ value for absolute agreement was −0.19 (95% CI: −2.17 to 0.53, *p* = 0.64), still indicating no agreement between sessions. See Table 4 for the comparison of test–retest PPB reliability results for left and right side and for the right side after the removal of outliers.

## Discussion

The aim of this study was to assess the test–retest reliability of PPB generated from sensory nerves of the lower back.

During the course of the study, it became imperative to explore the difference between PPB of a protocol using 40 and 50 ms ISIs to ensure the protocol was assessing PPB instead of two single-pulse SEP traces in succession. As no previous studies have investigated this question in any people with back pain or with a solely sensory nerve stimulation, data for ISIs stemming from mixed nerves, such as the median nerve used by Lenz et al. (2012), could not be used as reference values. It was shown that because of the substantial intra- and inter-individual variability, the differences in 40 and 50 ms ISI PPB did not reach statistical significance. Thus, it could be assumed that true PPB was assessed instead of two successive single-pulse SEP traces with the second cortical response being independent from the first.

The assessment of PPB by means of the present protocol did not demonstrate systematic differences between session 1 and 2 and hence did not show any evidence of order effects (Nevill and Atkinson, 1998).

However, a considerable amount of random error within the current PPB measurement protocol was demonstrated [CV for right side stimulation (95% CI) = 161.04% (123.21 to 232.56); CV for left side stimulation (95% CI) = 88.51% (67.71 to 127.81)]. With 95% LOA of ±3.55 for right side stimulation, it can be estimated that a healthy individual could change, in a worst-case scenario, between PPB values of −4.00, demonstrating PPS, and 3.11, indicating paired pulse disinhibition or facilitation. This variability could occur due to normal variation with this measurement protocol, clearly questioning its usefulness for research purposes. Especially, this high amount of normal variability could hamper comparisons between healthy individuals and people with expected alterations in cortical excitability, such as CLBP.

One reason for the high variability of results could have been the imbalance between male and female participants in our study sample, as 76% (*n* = 23) of the participants were female and only 24% (*n* = 7) male. There is evidence for gender specific differences in cortical SEPs generated from mixed nerves in that women have been shown to present with shorter latency values (Pelliccioni et al., 2014). Moreover, it was shown that PPS was significantly reduced subject to high estradiol levels throughout the menstrual cycle (Schloemer et al., 2020). However, using the current data, an unpaired *t*-test for session 1 PPB data showed no significant difference between male and female participants. The mean scores for right side PPB obtained in session 1 were 0.58 (SD = 0.39) for females and 0.89 (SD = 0.86) for males, [mean difference −0.31 (95% CI: −0.83 to 0.22, *p* = 0.10)]. The mean scores for left side PPB obtained in session 2 was 0.70 (SD = 0.65) for females and 0.60 (SD = 4.45) for males, [mean difference 0.11 (95% CI: −0.45 to 0.67, *p* = 0.70)]. Hence, the presentation of PPB as combined values for males and females would appear reasonable for the present study sample. However, despite the lack of significance, gender differences cannot be finally ruled out, because of the imbalanced sample size.

One potential contribution to the high random variability could have been the small sample size. The sample size target was initially based on sample size recommendations from the American Clinical Neurophysiology Society (American Clinical Neurophysiology Society, 2006a), which recommended a number of 30 healthy individuals for the establishment of laboratory-specific reference data, to which further data from clinical samples could then be compared. However, the numbers are lower than recommended for reliability studies. The final sample size for analysis was below the recommendations of 40 participants for reliability studies within the literature (Altman, 1991) due to personal and time resource constraints. A larger sample size might have led to more precise interval estimates (smaller confidence intervals). However, the sample size achieved is close to the recommended number and somewhat larger than those in other related studies investigating PPB (Hoeffken et al., 2010; Gatica Tossi et al., 2013; Enax-Krumova et al., 2017).

Still, we used the right side PPB reliability values *post hoc* as a basis for a power calculation (G*Power, Version 3.1.9.7, Franz Faul, University of Kiel, Kiel, Germany). With a mean session difference of −0.44 and a SD of session differences of 1.81, it could be estimated that 185 participants would be required for a future two-arm randomized controlled trial with PPB assessed *via* the current protocol as the outcome of interest. This sample size could be very difficult to achieve in a rehabilitation research context.

The high level of random variability could also potentially be attributed to elements of the measurement protocol itself, such as the stimulation intensity. As sensory nerves were stimulated, the stimulation intensity of the current protocol was set at the three-fold sensory threshold, as recommended by international guidelines (American Clinical Neurophysiology Society, 2006b). Brown et al. (2017) investigated the reliability of single-pulse SEPs generated from stimulation of the median nerve. They found that SEP amplitudes (N20/P25) with a stimulation intensity at motor threshold and 150% of the motor threshold showed an ICC value of 0.91 for both, indicating high reliability. In addition, N20 latency values showed an ICC score of 0.90, likewise indicating high reliability. The authors concluded that the most reliable measures of latencies and amplitudes were gathered at higher stimulation intensities. They explained this feature by the fact that measures with high reliability might evoke physiologically maximal responses in each individual and may therefore have a favorable signal-to-noise ratio, making it easier to detect small differences between sessions.

In this study, it was decided to assess sensory nerves of the lower back, which needed to be stimulated with a predefined low intensity to prevent any muscle contractions, which could have blurred or superposed the recorded signal. This may have resulted in the signal not being sufficiently contrasted with the background noise to distinguish the recorded subtle SEP signal of interest. However, all subjects were able to perceive the applied stimulus intensities and reported them as, e.g., throbbing, pricking or galloping. Although higher stimulation intensities would not have fitted into the concept of sole sensory nerve stimulation, as used in this study, this contradiction in terms could have been solved by choosing a mixed nerve, such as the median nerve. This would have allowed for higher stimulation intensities. Early research regarding the optimal intensity for median nerve stimulation demonstrated that the amplitude of cortical responses significantly increased when the stimulus intensity was increased from sensory threshold to the first muscle twitch of the thumb (Hume and Cant, 1978), potentially leading to more distinguishable SEP signals, which could have led to more reliable results.

In addition, the computation of PPS involves inhibition generated from intracortical networks, which also requires an interplay of multiple ascending, descending and local neuronal signals (Pais-Vieira et al., 2013). Hence, PPB could be seen as a more complex and distributed pattern of neuronal cortical activity compared to a single-pulse SEP signal, although its underlying mechanisms are still not fully understood (Lenz et al., 2012).

Moreover, the magnitude of the ascending somatosensory signal elicited by stimulation of cutaneous sensory nerves compared to stimulation of mixed nerves might have contributed to the poor reliability results. By default, cutaneous sensory nerve SEPs show a lower amplitude than those created by stimulation of mixed nerves, such as the median nerve, because fewer fibers are excited (Watson and Carter, 2016). Hence, stimulating the median nerve elicits higher amplitudes making the phenomenon of PPB easier to detect compared to stimulating cutaneous sensory nerves against the considerable amount of random intrinsic electrical fluctuations associated with the recording of a SEP signals (Lim et al., 2012; Stude et al., 2016).

However, as the aim of this study was to assess the test–retest reliability of cortical excitability by SEPs generated from the bodily area of interest, in the lower back, only cutaneous sensory nerves qualified for this purpose. Using segmental or dermatomal SEPs generated from lumbar spinal nerves would not have qualified for assessing PPB, as SEPs generated from these nerves provide information regarding nerve root compromises (Chabot et al., 1995; Chiappa, 1997a), which would be termed as a specific back pain disorder (Waddell, 2004).

Onishi et al. (2018) evaluated the test–retest reliability of PPB after median nerve stimulation by means of Magnetoencephalography (MEG). ICC values for the pooled data of mN20 PPB ratio demonstrated no statistically significant correlation (ICC = −0.045, *p* = 0.634) whereas excellent reliability data for the P35m_PPD ratio (ICC = 0.76, *p* = 4.44 × 10^–11^) were reported. Although PPB was measured by a different approach, the data mirror the inherent substantial variability of the construct under investigation and demonstrate the necessity to investigate the reliability of any measurement approach claiming to assess PPB, which is in keeping with the findings of this study.

Lim et al. (2015) investigated cortical excitability of S1 and its potential role in clinical pain in 17 female patients with FM and 21 healthy controls. The authors used a paired-pulse median nerve stimulation and MEG to quantify PPS in both groups. The authors reported, that the amplitudes of the second response were markedly suppressed as compared with the first response in both hemispheres in healthy subjects but intracortical inhibition in the S1 was compromised bilaterally in the patient group which suggested that changes of cortical inhibition of S1 may contribute to the pathophysiology of FM pain. Although our findings were based upon the assessment of sensory nerves of the lower back as that was the focus of the study, an approach worth pursuing in future studies could be the assessment of mixed nerves, such as the median nerve, in patients with CLBP.

In conclusion, this study presents a protocol and associated normative data for assessing single-pulse SEPs and PPB generated from sensory nerves of the lower back in healthy individuals. The test–retest reliability of the protocol was found to be very poor. By default, this also questions the validity of the protocol, as reliability is seen as the bedrock of clinimetric properties on which any further investigation is based (Bennett and Miller, 2010). This finding has wider implications for PPB protocols as no other study has published test–retest reliability data for PPB procedures. If these findings were replicated in other groups and other nerves it would question the validity of this measure more generally. However, these findings are restricted to healthy people and the sensory nerves of the lower back and may not be generalizable.

## Data availability statement

The raw data supporting the conclusions of this article will be made available by the authors, without undue reservation.

## Ethics statement

The studies involving human participants were reviewed and approved by Teesside University’s School of Health and Social Care Research Governance and Ethics Board (Study No. 015/16) and the Ethics Committee of the German National Physiotherapists Society. The patients/participants provided their written informed consent to participate in this study.

## Author contributions

KE, CR, CG, and DM considerably contributed to the study conceptualization. KE, CR, CG, HD, and VM contributed to the development of the study design and formal analysis, and contributed to data curation and formal analysis. KE performed all investigations. CG and HD contributed to provision of study materials and software. KE and CR drafted the initial manuscript. All authors critically revised and edited the manuscript for important intellectual content and approved the final version.
