# Supplementary material for: Low test–retest reliability of a protocol for assessing somatosensory cortex excitability generated from sensory nerves of the lower back
Source: Front Hum Neurosci. 2022 Aug 23;16:898759. doi: 10.3389/fnhum.2022.898759 (PMC9445117; doi:10.3389/fnhum.2022.898759)
Supplement: Supplementary file 1 [file Data_Sheet_1.docx]

Supplementary Material

# Supplementary Tables

## GRRAS checklist for reporting of studies of reliability and agreement

Version based on Table I in: Kottner J, Audigé L, Brorson S, Donner A, Gajeweski BJ, Hróbjartsson A, Robersts C, Shoukri M, Streiner DL. Guidelines for reporting reliability and agreement studies (GRRAS) were proposed. J Clin Epidemiol. 2011;64(1):96-106

| **Section** | **Item**  **#** | **Checklist item** | **Reported on page #** |
| --- | --- | --- | --- |
| Title/Abstract | 1 | Identify in title or abstract that interrater/intrarater reliability or agreement was investigated. | 2 |
| Introduction | 2 | Name and describe the diagnostic or measurement device of interest explicitly. | 5 |
|  | 3 | Specify the subject population of interest. | 4 |
|  | 4 | Specify the rater population of interest (if applicable). | n.a. |
|  | 5 | Describe what is already known about reliability and agreement and provide a rationale for the study (if applicable). | 3-4 |
| Methods | 6 | Explain how the sample size was chosen. State the determined number of raters, subjects/objects, and replicate observations. | 6/7 |
|  | 7 | Describe the sampling method. | 4 |
|  | 8 | Describe the measurement/rating process (e.g. time interval between repeated measurements, availability of clinical information, blinding). | 5-6 |
|  | 9 | State whether measurements/ratings were conducted independently. | 5 |
|  | 10 | Describe the statistical analysis. | 7 |
| Results | 11 | State the actual number of raters and subjects/objects which were included and the number of replicate observations which were conducted. | 8 |
|  | 12 | Describe the sample characteristics of raters and subjects (e.g. training, experience). | 8 |
|  | 13 | Report estimates of reliability and agreement including measures of statistical uncertainty. | 9 |
| Discussion | 14 | Discuss the practical relevance of results. | 11 |
| Auxiliary material | 15 | Provide detailed results if possible (e.g. online). | Supplememtary Material 2/3 |

## 1.2 Descriptive data for latencies, amplitudes and side-to-side differences for single SEPs after stimulation of the sural nerve

|  |  |  | **Mean** | **SD** | **Median** | **IQR** | **Range** |
| --- | --- | --- | --- | --- | --- | --- | --- |
| Latencies (ms) | right side stimulation | N30 | 34.53 | 3.62 | 34.20 | 32.38 – 36.25 | 24.20 – 43.80 |
|  |  | P40 | 42.85 | 3.74 | 42.05 | 40.13 – 45.38 | 36.50 – 53.20 |
|  |  | P40-height  adjusted | 24.92 | 1.94 | 24.35 | 23.58 – 25.65 | 21.60 – 30.60 |
|  |  | N50 | 53.03 | 3.55 | 52.35 | 50.50 – 54.90 | 46.60 – 64.30 |
|  | left side stimulation | N30 | 33.98 | 3.79 | 33.35 | 31.83 – 36.23 | 26.40 – 44.70 |
|  |  | P40 | 42.52 | 4.40 | 42.00 | 39.60 – 44.73 | 30.60 – 54.50 |
|  |  | P40-height adjusted | 24.75 | 2.47 | 24.50 | 23.68 – 44.73 | 16.70 – 31.30 |
|  |  | N50 | 52.83 | 5.07 | 52.60 | 50.38 – 55.10 | 37.30 – 68.40 |
| Amplitudes (µV) | right side stimulation | pair 1 (N30/P40) | 1.51 | 0.77 | 1.35 | 0.97 – 2.00 | 0.27 – 3.35 |
|  |  | pair 2 (P40/N50) | 2.66 | 1.48 | 2.29 | 1.75 – 3.42 | 0.42 – 5.97 |
|  | left side stimulation | pair 1 (N30/P40) | 1.70 | 1.09 | 1.44 | 0.93 – 2.06 | 0.31 – 5.18 |
|  |  | pair 2 (P40/N50) | 2.94 | 1.95 | 2.69 | 1.54 – 3.63 | 0.23 – 8.36 |
| Latencies (ms) | Side-to-side differences (STSD) | N30 | -0.55 | 3.40 | -0.30 | -1.20 – 0.45 | -12.50 – 9.60 |
|  |  | P40 | -0.32 | 4.01 | 0.05 | -0.80 – 1.33 | -23.00 – 2.70 |
|  |  | P40-height adjusted | -0.17 | 2.23 | 0.03 | -0.48 – 0.75 | -10.38 – 3.06 |
|  |  | N50 | -0.20 | 3.83 | 0.35 | -1.30 – 1.48 | -16.30 – 4.40 |
| Amplitudes (µV) | Side-to-side asymmetry  ratios (STSAR) of amplitudes | pair 1 (N30/P40) | 0.50 | 0.41 | 0.37 | 0.20 – 0.76 | 0.02 – 1.49 |
|  |  | pair 2 (P40/N50) | 0.45 | 0.32 | 0.41 | 0.20 – 0.60 | 0.02 – 1.64 |

Legend: IQR = interquartile range; ms = milliseconds; µV = microvolt; height adjustment followed the formula by Joerg (1983)

## 1.3 Histograms, Q-Q-plots and box plots of change scores for PPB after stimulation of lower back sensory nerves

| **Parameter** | **Histograms** | **Normal Q-Q-Plots** | **Box Plots** |
| --- | --- | --- | --- |
| **PPB right side** | 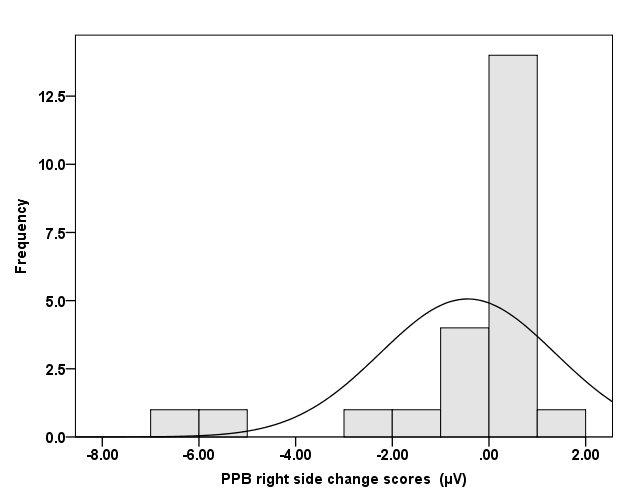 | 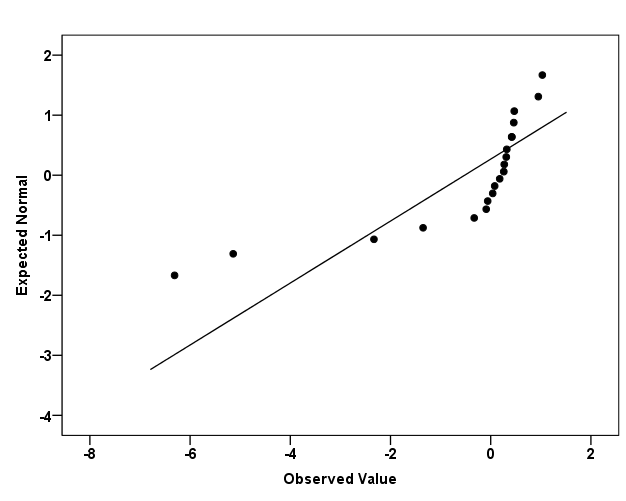 | 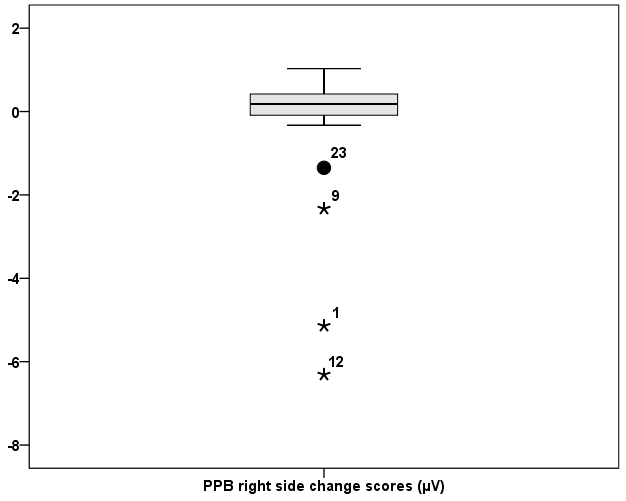 |
| **PPB right side – outliers removed** | 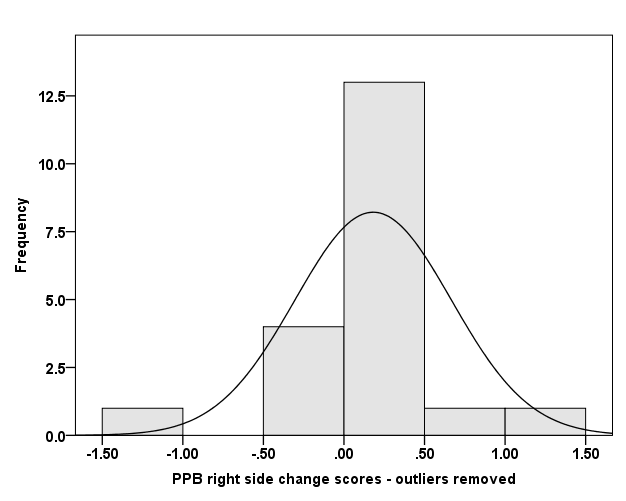 | 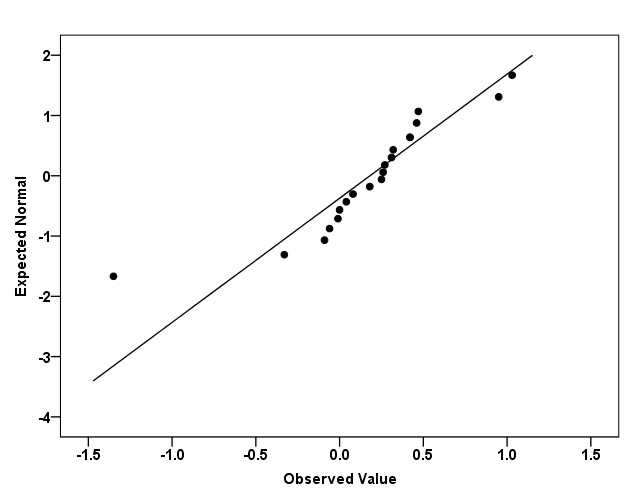 | 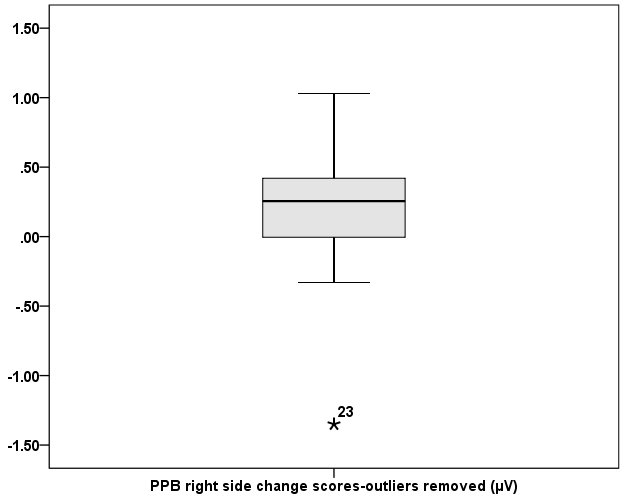 |
| **PPB left side** | 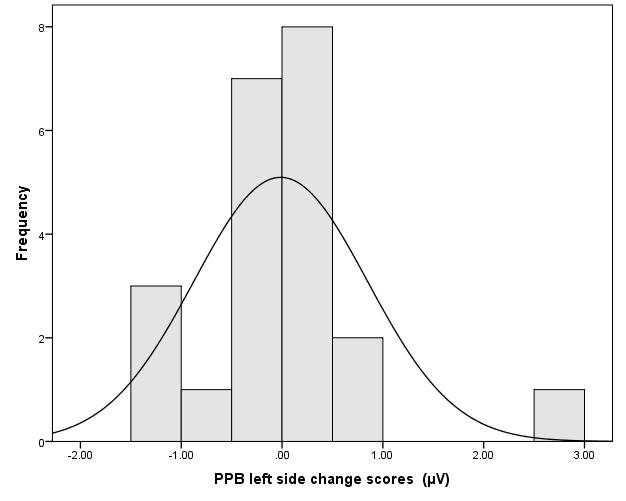 | 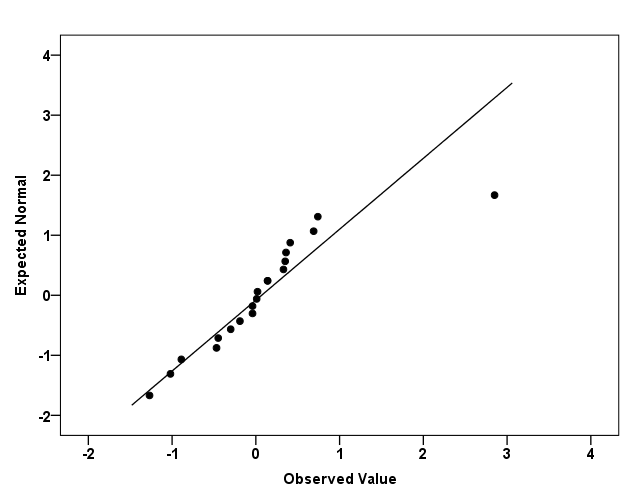 | 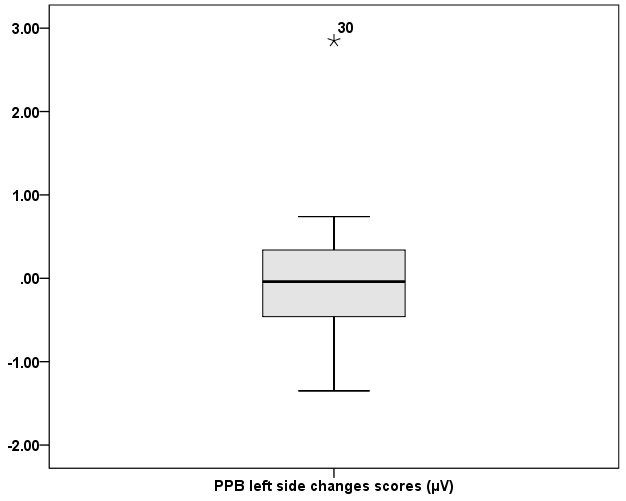 |

*Legend: PPB = paired-pulse behaviour; µV = Microvolt; right/left indicates stimulation side;*

*Note: the asterix in the box blots denotes values exceeding 3*interquartile range (IQR), the dot indicates values >1.5*IQR*
